# WHO cone bio-assays of classical and new-generation long-lasting insecticidal nets call for innovative insecticides targeting the knock-down resistance mechanism in Benin

**DOI:** 10.1186/s12936-017-1727-x

**Published:** 2017-02-15

**Authors:** Marius Allossogbe, Virgile Gnanguenon, Boulais Yovogan, Bruno Akinro, Rodrigue Anagonou, Fiacre Agossa, André Houtoukpe, Germain Gil Padonou, Martin Akogbeto

**Affiliations:** 1 Centre de Recherche Entomologique de Cotonou (CREC), Cotonou, Benin; 2 Université d’Abomey-Calavi, Abomey-Calavi, Benin; 3 Medical Care and Development International, Washington, USA

**Keywords:** LLINs, Bio-efficacy, Piperonyl butoxide, Resistant mosquitoes

## Abstract

**Background:**

To increase the effectiveness of insecticide-treated nets (ITN) in areas of high resistance, new long-lasting insecticidal nets (LLINs) called new-generation nets have been developed. These nets are treated with the piperonyl butoxide (PBO) synergist which inhibit the action of detoxification enzymes. The effectiveness of the new-generation nets has been proven in some studies, but their specific effect on mosquitoes carrying detoxification enzymes and those carrying both detoxification enzymes and the knock-down resistance gene in Benin is not well known. Thus, the objective of this study is to evaluate the efficacy of LLINs treated with PBO on multi-resistant *Anopheles gambiae* s.l.

**Methods:**

The study occurred in seven cities in Benin, Abomey, Cotonou, Porto-Novo, Zangnanado, Parakou, Malanville and Tanguiéta, and included ten locations selected on a north–south transect. Mosquito larvae were collected from these sites, and adult females from these larvae were exposed to single-pyrethroid-treated nets (LifeNet, PermaNet 2.0, Olyset Net) and bi-treated nets (PermaNet 3.0 and Olyset Plus) based on their level of resistance and using WHO cone tests following WHO guidelines.

**Results:**

The different LLINs showed 100% mortality of the susceptible laboratory strain Kisumu and the resistant strain Ace-1R Kisumu. However, with the resistant laboratory strain kdr-Kisumu, mortality was low (16–32%) for all LLINs except PermaNet 3.0 (82.9%). The mortality of local strains carrying only the *kdr* mechanism varied from 0 to 47% for the single-pyrethroid-treated LLINs and 9 to 86% for bi-treated LLINs. With local strains carrying several mechanisms of resistance (kdr + detoxification enzymes), the observed mortality with different LLINs was also low except for PermaNet 3.0, which induced significantly higher mortality, usually greater than 75% (p < 0.001), with multi-resistant strains. The inhibition of the mortalities induced by the LLINs (11–96%) on multi-resistant field populations was similar to the inhibition observed with the laboratory strain carrying only the knock-down resistance mechanism (kdr-Kisumu) (p > 0.05).

**Conclusion:**

This study showed that the new-generation LLINs treated with pyrethroids and PBO showed better efficacy compared to conventional LLINs. Although the addition of PBO significantly increased the mortality of mosquitoes, the significant role of the *kdr* resistance gene in the low efficacy of LLINs calls for LLIN technology innovation that specifically targets this mechanism.

## Background

Malaria is a major public health problem worldwide, and particularly so in Benin. It remains a permanent threat from its high morbidity (214 million) and mortality (438,000). Africa is the most endemic region affected (395,000 deaths per year) [1]. It affects one-fifth of the world population. However, this proportion has decreased significantly by 37% between 2000 and 2015 due to the effect of malaria prevention and treatment methods, including long-lasting insecticidal nets (LLINs), indoor residual spraying of residual insecticides (IRS), chemo-prevention for pregnant women and children, and therapeutic treatment with artemisinin-based combinations.

Among these prevention methods, LLINs have emerged in recent years as a privileged tool to prevent malaria. The insecticides selected by the World Health Organization (WHO) for LLIN treatment are pyrethroids, which have little toxicity to humans, are effective at low doses, are fast acting (knock-down effect) and, along with repellants, have an irritant effect [2]. The Abuja Conference, which brought together all the leaders of Africa and other UN representative states, donors and NGOs in April 2000, gave impetus to a political commitment to the fight against malaria with the use of insecticide treated nets (ITNs) [3]. Efforts are being made to increase accessibility for populations, especially pregnant women and children under five, who are vulnerable to malaria, a major cause of perinatal mortality, low birth weight and maternal anaemia [1].

Several research studies have been conducted and have shown the effectiveness of ITNs in the fight against malaria in Burkina Faso [4], Cameroon [5], Gambia [6–9], the Democratic Republic of Congo [10], Kenya [11], Ghana [12], Benin [13] and Côte d’Ivoire [14].

However, several studies have shown that *Anopheles gambiae* s.l. has developed strong resistance to pyrethroids and DDT in Benin, with a very high knock-down resistance frequency of approximately 80% in the urban areas of Cotonou and in rural areas [15–23].

Despite this resistance developed by *An. gambiae* s.l. to pyrethroids, LLINs remain effective in vector resistance areas [24] and provide protection through their mechanical barrier role [25]. However, Asidi et al. [26] showed a decrease in their effectiveness in areas of high resistance of Anopheles in southern Benin. Major developed resistance mechanisms are the targets of modification (*kdr* resistance and *ace*-*1R*) and metabolic resistance (over-expression of detoxification enzymes, oxidases, esterases, GST) [27]. The *kdr* mutation is associated with pyrethroid and DDT resistance, and *ace*-*1R* is associated with organophosphate and carbamate resistance (two classes of insecticides which are not used to treat LLINs) [15, 28].

To increase the effectiveness of ITNs in areas of high resistance, new nets treated with a so-called new-generation of chemicals has been developed. They are treated with a synergist called piperonyl butoxide (PBO). For some LLINs, the PBO is used on all sides of the net (Olyset Plus^®^). For others, only the upper part of the net is processed (PermaNet^®^ 3.0). The principle of an ITN synergist is to inhibit the action of detoxification enzymes, which will result in increasing the effectiveness of the insecticide against resistant populations of mosquitoes.

Evidence of the efficacy of PermaNet 3.0 has been shown in some studies, particularly in Tanzania [29], but we do not know its specific action on mosquitoes carrying detoxification enzymes and on those carrying both detoxification and *kdr* mechanisms in West Africa, particularly in Benin. There have been limited data on the bio-efficacy of new-generation LLINs against multi-resistant mosquitoes in Africa in general and particularly in Benin. Thus, the objective of this study is to evaluate the efficacy of long-lasting insecticidal nets (LLINs) treated with PBO on multi-resistant *An. gambiae* s.l. populations in Benin. It aims to assess the bio-efficacy of LLINs in areas with a high frequency of molecular resistance genes (*kdr* and *ace*-*1R*) and over-expression of detoxification enzymes (oxidases, esterases, GST). The efficacy of the new-generation LLINs against pyrethroid-resistant Anopheles was also compared to that of conventional LLINs.

## Methods

### Study design

This study is transversal and compares variability of the efficacy of two different types of LLINs against *An. gambiae* s.l. carrying *kdr* resistance mutations and detoxification enzymes in Benin. The two types of LLINs included conventional LLINs only treated with pyrethroids (Olyset Net, LifeNet, and PermaNet 2.0) and a second type of new-generation LLIN treated with pyrethroids and piperonyl butoxide (PBO), which inhibits the action of enzymes, particularly oxidases.

The study was conducted in Benin, a West African country from June 2015 to March 2016. Among the 12 departments of Benin surveyed, seven were selected in this study (Atlantique, Littoral, Oueme, Zou, Borgou, Atacora and Alibori). Priority was given to areas where higher oxidase activity was observed compared to the susceptible strain *An. gambiae* Kisumu. They were represented by Abomey, Cotonou, Porto-Novo, Zangnanado, Parakou, Malanville and Tanguiéta districts. The assessment of oxidase activity was conducted on 50 *An. gambiae* s.l. collected from each district using haem-peroxidase assay as described by Brogdon et al. [30].

The larvae of these mosquito populations were collected in different ecological areas (vegetable, urban, rice and cotton areas). The study was also conducted on resistant laboratory strains (kdr-Kisumu and ace-1R-Kisumu).

### Study sites

#### Malanville

**Fig. 1:**
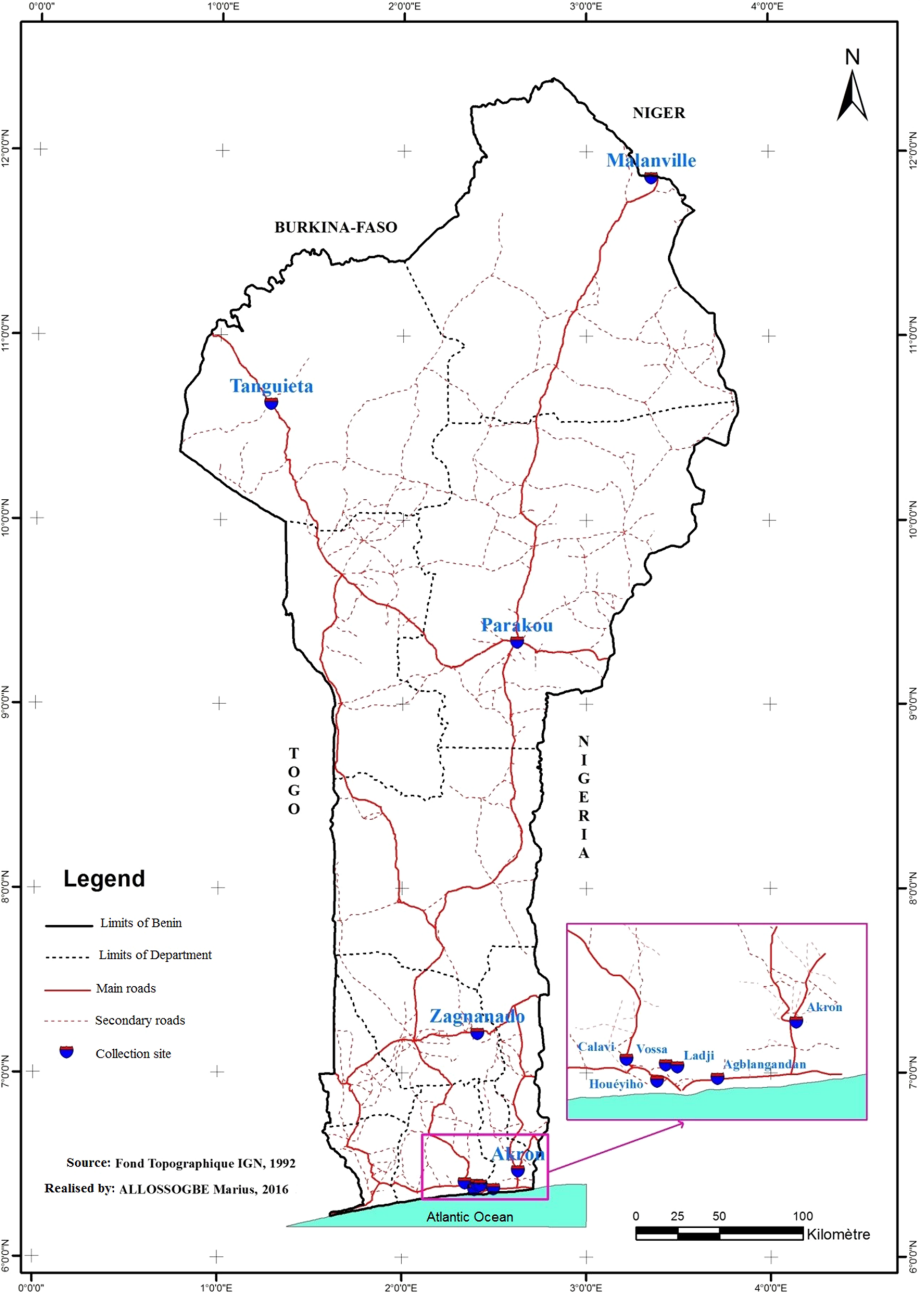
Map of Benin showing the study locations

Malanville district is bordered on the north by the Republic of Niger, on the south by Kandi and Segbana districts, on the west by Karimama district and on the east by the Republic of Nigeria. It has an area of 3016 km^2^ and had a population of 144,843 inhabitants in 2013 (Fig. 1).

#### Tanguieta

It is bordered on the north by the Republic of Burkina Faso, on the south by Boukoumbe district, on the east by Kerou, Kouande and Tounkountouna districts and on the west by Materi and Cobly districts. It covers an area of 5456 km^2^ and had a population of 77,987 inhabitants in 2013 (Fig. 1).

#### Abomey-Calavi

Abomey-Calavi is bounded on the north by Ze district, on the south by the Atlantic Ocean, on the east by Cotonou and So-Ava districts, and on the west Ouidah and Tori-Bossito districts. It has an area of 539 km^2^ and had a population of 438,564 inhabitants in 2013 (Fig. 1).

#### Cotonou

Cotonou is bordered on the North by So-Ava district and Nokoue lake, on the south by the Atlantic Ocean, on the east by Seme-Podji and on the west by Abomey-Calavi district. It has an area of 79 km^2^ and had a population of 947,917 inhabitants in 2013 (Fig. 1).

#### Porto-Novo

Porto-Novo is bounded on the north by Akpro-Missérete and Avrankou districts, on the south by Seme-Podji, on the west by Aguegues district and on the east by Adjarra district. It covers an area of 223,552 km^2^ and had a population of 318,608 inhabitants in 2013 (Fig. 1).

#### Parakou

It is bordered on the north by N’Dali district and on the south, east and west by Tchaourou district; it has an area of 441 km^2^ and had a population of 213,498 inhabitants in 2013 (Fig. 1).

#### Zangnanado

This town is bounded on the north by Dassa-Zoume district, on the south by Ouinhi and Zogbodomey districts, on the west by Cove, Zakpota and Djidja districts and on the east by Ketou and Adja-Ouere. It has an area of 540 km^2^ and had a population of 52,387 inhabitants in 2013 (Fig. 1).

### Larvae collection

Bio-efficacy tests were conducted at various selected sites. Such tests required mosquitoes of 2–5 days old, so the larvae were collected. These collections were conducted in the different localities mentioned above. *Anopheles gambiae* s.l. larvae and pupae were collected from different locations at each site and carried to the insectarium of the Entomological Research Center of Cotonou (CREC), where they were reared to adult stage at a relative humidity of 70–80% and a temperature of 25–30 °C. Female adults aged 2–5 days were used for bio-efficacy tests.

### Highlighting resistance mechanisms

Before the bioassays, living and dead mosquito populations kept after susceptibility testing were analyzed by PCR to detect the genotypes of the *kdr* gene. The detection of *kdr* mutation L1014F was performed according to the method of Martinez-Torres et al. [31].

For the molecular characterization of insecticide resistance, two molecular markers were used for characterization of the resistance genes, *kdr* and *ace*-*1R*.

Similarly, for the biochemical characterization of resistance mechanisms, biochemical assays were performed to compare the activity levels of mixed function oxidases (MFO), non-specific esterases (NSE) and glutathione S-transferases (GST) according to the protocol described by Hemingway et al. [32] in susceptible Kisumu and field *An. gambiae* strains. The mosquitoes used for biochemical analysis had not been exposed to insecticides before the biochemical assessment. These enzyme activities were measured using a sample of 50 mosquitoes per site.

### Mosquito nets

Five types of long-lasting insecticidal nets were evaluated in this study. The group of mono-treated LLINs included LifeNet (polypropylene LLIN with fiber coated with 340 mg/m^2^ ± 25% deltamethrin), Olyset Net (polyethylene LLIN with permethrin incorporated into the fibers at 20 ± 3 g/kg), and PermaNet 2.0 (polyester LLIN with fiber coated with deltamethrin at 55 mg/m^2^ ± 25%). The group of new-generation LLINs included: Olyset Plus (same characteristics as Olyset Net but with PBO incorporated throughout the LLIN) and PermaNet 3.0 (polyethylene roof with deltamethrin at 2.8 g/kg ± 25% and PBO at 4.0 g/kg ± 25% incorporated into the fibers, and polyester lateral sides with the fibers coated with deltamethrin at 2.8 g/kg ± 25%). All these nets were obtained from local markets. All nets included in the study are rectangular and were selected by type.

### Cone test

The cone test is used to assess the effectiveness of an insecticide and its persistence on the net. It was conducted following the WHO protocol. This test aims to compare the behaviour of mosquitoes while in contact with treated mosquito nets without PBO or with PBO.

Cone tests were performed on five types of nets (Olyset Plus, Olyset Net, LifeNet, PermaNet 2.0 and PermaNet 3.0). These tests were carried out using fragments of LLINs (30 cm × 30 cm) cut from five (05) positions on each net. Two standard cones were fixed with a plastic sheet on each of the five (05) screen fragments. For PermaNet 3.0 LLIN, an additional two cones were added on the PBO-containing roof. Five unfed *An. gambiae* females aged 2–5 days (Kisumu or wild type) were introduced into each cone placed on the LLIN for 3 min. After exposure, the mosquitoes were removed from the cones using a mouth aspirator and then transferred into paper cups and provided 10% sugar solution. Mosquito knock-down was recorded every 5 min for 60 min. A negative control (untreated net) was included in each series of cone tests. After 24 h of observation, mortality post exposure was recorded. No correction of mortality with Abbott’s formula was used as mortality in the control was <5%. All these operations were carried out at a temperature of 25 ± 2 °C and a humidity of 70 ± 10%.

### Data analysis

According to the WHO, the bio-effectiveness threshold is 95% knock-down and 80% mortality for laboratory mosquitoes; but for resistant field mosquito populations, we used a threshold of 70% knock-down and 50% mortality. Therefore, all nets showing less than 95% knockdown for laboratory mosquitoes and 70% for field mosquitoes after 60 min, or less than 50% mortality for laboratory mosquitoes and 50% for field mosquitoes after 24 h of observation, were considered ineffective. These knock-down thresholds were chosen taking into account the *kdr* resistance level observed in the country in general (>50%).

The inhibition of mortality induced by resistance mechanisms was estimated using the following equation:

$$ {\text{Inhibition }} = \, 1 - \, \left( {{\text{p}}1 \, /{\text{ p}}2} \right) \, \times \, 100 $$

where p1 = proportion of resistant mosquitoes dead and p2 = proportion of susceptible Kisumu mosquitoes dead.

To determine if there was any significance difference between the outcome variables (knock-down, mortality and inhibition), Poisson regression (for numeric data) and logistic regression (for proportional data) were used. The 50 and 95% knock-down times and their confidence intervals were obtained after log-probit regression using the method described by Finney [33].

## Results

### Characteristics of the studied mosquito populations

The majority of female mosquitoes were collected and identified morphologically as *An. gambiae* s.l. The biochemical and molecular analyses indicated that among ten sites, five showed significantly higher oxidase activity than the susceptible strain Kisumu (Table 1). Esterases were significantly expressed in the Tanguieta mosquito population (Table 1). Over-expression of glutathione-S-transferase was observed at four sites (Table 1). However, the allelic frequency of the *kdr* mutation was high at almost all sites and ranged from 0.03 to 0.93.

**Table 1:** Biochemical and molecular characteristics of the *Anopheles gambiae* s.l. populations tested

Strains of *An. gambiae* s.l.	Average oxidase activity (min/mg protein)	Average α esterase activity (min/mg protein)	Average β esterase activity (min/mg protein)	Average glutathione-S-transferase activity (min/mg protein)	*kdr* frequency
Kisumu	0.1015^a^	0.07409^a^	0.07655^a^	0.3846^a^	0^a^
Agblangandan	0.07966^a^	0.07883^a^	0.06117^a^	0.7319^b^	0.03^a^
Abomey-Calavi	0.08454^a^	0.07149^a^	0.05929^a^	0.4295^a^	*0.93* ^b^
Akron	0.1604^b^	0.08589^a^	0.07897^a^	2.221^b^	*0.74* ^b^
Houeyiho	0.17.39^b^	0.07694^a^	0.08774^a^	0.4042^a^	*0.9* ^b^
Vossa	0.07566^a^	0.06897^a^	0.06389^a^	0.7078^a^	*0.84* ^b^
Ladji	0.1737^b^	0.07146^a^	0.0774^a^	1.194^b^	*0.92* ^b^
Bame	0.1106^a^	0.0588^a^	0.06223^a^	0.2901^a^	0.78^b^
Malanville	0.06549^a^	0.04949^a^	0.04871^a^	0.1723^a^	*0.90* ^b^
Parakou	0.1536^b^	0.08124^a^	0.08871^a^	0.4698^a^	*0.74* ^b^
Tanguieta	0.2267^b^	0.1585^b^	0.1442^b^	1.182^b^	*0.85* ^b^

^a, b^Values with the same superscript do not differ significantly at α = 0.05

### Knock-down (KD) and mortality of laboratory strains

**Fig. 2:**
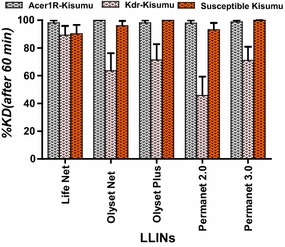
Mosquito knock-down at 60 min post-exposure to LLINs

Figure 2 shows the proportion of laboratory mosquitoes (ace-1R-Kisumu, kdr-Kisumu, and susceptible Kisumu) knocked down after 60 min for each LLIN. The Olyset Plus and PermaNet 3.0 LLINs induced 100% knock-down of *An. gambiae* Kisumu. The knock-down effect was 96.15% for Olyset, 90.2% for LifeNet and 93.22% for PermaNet 2.0.

With the ace-1R-Kisumu strain, which carries the acetylcholinesterase-1 resistance gene, there was a knock-down effect greater than 95% for all nets, with 98.11% for LifeNet, 100% for Olyset, 98.18% for Olyset Plus, 97.96% for PermaNet 2.0, and 98.78% for PermaNet 3.0 (Fig. 2).

For the kdr-Kisumu strain (carrying the resistance knock-down), the knock-down effects observed were 89.29% for LifeNet, 63.64 for Olyset Net, 71.43% for Olyset Plus, 45.78 for PermaNet 2.0 and 71.05% for PermaNet 3.0 (Fig. 2).

**Fig. 3:**
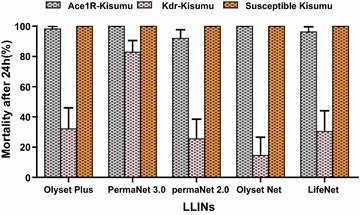
Mosquito mortalities 24 h post-exposure to LLINs

Kisumu and ace-1R-Kisumu (Fig. 3). With the kdr-Kisumu strain, mortality was 16% for Olyset Net, 26% for PermaNet 2.0, 28% for LifeNet, and 32.1% for Olyset Plus but was more than 82.9% for PermaNet 3.0. Therefore, based on the bio-efficacy threshold set by WHO (80%), PermaNet 3.0 was effective on all laboratory strains, and Olyset Plus was only effective on the susceptible and ace-R1-Kisumu strains (Fig. 3).

### Inhibition of mortality conferred by the *kdr* resistance gene

Comparing the mortality observed with the susceptible Kisumu strain with that of the resistant kdr-Kisumu strain, the inhibition of mortality induced by the *kdr* gene regarding the effectiveness of LLINs was 84% for Olyset Net, 74% for PermaNet 2.0, 72% for LifeNet, 68% for Olyset Plus and 17% for PermaNet 3.0.

### Knock-down (Kd) effect and mortality induced by mosquito nets on local *An. gambiae* s.l.

Approximately 2819 local *An. gambiae* s.l. mosquitoes and 889 *An. gambiae* Kisumu laboratory strain mosquitoes were tested on different types of LLINs. Tables 2 and 3 show the percentage of local strain mosquitoes knocked down after 60 min for LifeNet, Olyset Net, Olyset Plus, PermaNet 2.0, and PermaNet 3.0.

**Table 2:** Distribution of the knock-down rate observed in localities where there was only one resistance mechanism (*kdr*)

Strains	LLINs	N mosquito tested	KD after 60 min	95% CI	Mortality after 24 h (%)
Malanville	LifeNet	55	72.27	[59.03–83.86]	27.27
	Olyset Net	53	30.19	[18.34–44.34]	05.56
	Olyset Plus	51	54.9	[40.34–68.87]	21.56
	PermaNet 2.0	59	28.81	[17.76–42.08]	47.46
	PermaNet 3.0	84	95.24	[88.25–98.69]	61.90
Abomey-Calavi	LifeNet	53	9.43	[3.13–20.66]	7.54
	Olyset Net	54	11.11	[4.18–22.63]	5.56
	Olyset Plus	55	29.09	[17.62–49.90]	20
	PermaNet 2.0	52	70.49	[57.43–81.84]	26.92
	PermaNet 3.0	72	81.94	[71.1–90.02]	86.11
Zagnanado (Bamè)	LifeNet	58	68.97	[55.45–80.46]	10.34
	Olyset Net	54	23.08	[12.53–36.84]	00
	Olyset Plus	55	33.96	[21.51–46.27]	09.43
	PermaNet 2.0	53	52.83	[38.63–66.7]	03.77
	PermaNet 3.0	75	63.93	[57.61–79.47]	62.67
Vossa	LifeNet	54	62.96	[48.74–75.71]	20.37
	Olyset Net	57	21.05	[11.37–33.89]	14.03
	Olyset Plus	53	41.51	[28.13–55.87]	15.05
	PermaNet 2.0	51	52.94	[38.45–67.07]	23.52
	PermaNet 3.0	73	79.45	[68.38–88.02]	39.75

*N* number, *KD* knock-down, *min* minutes, *CI* confidence interval, *h* hours

**Table 3:** Distribution of the knock-down rate observed in localities where there were several resistance mechanisms (*kdr* + metabolic resistance)

Strains	LLINs	N mosquito tested	KD after 60 min	95% CI	Mortality (%)
Agblangandan	LifeNet	53	50.94	[36.83–64.96]	15.09
	Olyset Net	54	20.75	[10.84–34.11]	07.4
	Olyset Plus	55	50.91	[37.07–64.65]	34.72
	PermaNet 2.0	47	36.17	[22.67–51.58]	17.02
	PermaNet 3.0	66	60.61	[47.80–72.42]	65.15
Ladji	LifeNet	57	85.96	[74.2–93.74]	47.36
	Olyset Net	57	50.88	[37.28–64.37]	40.35
	Olyset Plus	56	42.86	[29.71–56.78]	41.07
	PermaNet 2.0	50	66	[51.23–78.79]	14
	PermaNet 3.0	69	88.41	[78.42–94.86]	44.93
Akron	LifeNet	52	30.77	[18.71–45.1]	15.38
	Olyset Net	54	31.48	[19.52–45.55]	5.56
	Olyset Plus	55	74.55	[60.99–85.33]	25.45
	PermaNet 2.0	61	70.49	[57.43–81.84]	54.09
	PermaNet 3.0	82	81.71	[71.63–89.38]	89.02
Parakou	LifeNet	51	43.14	[29.34–57.75]	09.80
	Olyset Net	52	26.92	[15.56–4 1.02]	07.69
	Olyset Plus	50	66	[51.23–78.79]	28
	Permanet 2.0	56	39.29	[26.49–53.25]	37.50
	Permanet 3.0	88	98.86	[93.83–99.97]	82.95
Houeyiho	LifeNet	54	61.11	[46.87–74.08]	14.81
	Olyset Net	52	23.08	[12.53–36.84]	3.84
	Olyset Plus	59	49.15	[35.89–62.5]	23.72
	Permanet 2.0	54	46.3	[32.62–60.39]	22.22
	Permanet 3.0	65	73.85	[61.46–83.97]	61.54
Tanguieta	LifeNet	–	–	–	–
	Olyset Net	–	–	–	–
	Olyset Plus	51	74.51	[60.36–85.67]	56.86
	PermaNet 2.0	62	75.81	[63.25–85.78]	32.26
	PermaNet 3.0	86	100	[88.78–100]	78.82

*N* number, *KD* knock-down, *min* minutes, *CI* confidence interval, *h* hours

### Knock-down (KD) and mortality induced by the LLINs on mono-resistance mosquito strains

Only PermaNet 3.0, Olyset Plus and LifeNet LLINs showed a knock-down effect greater than 50% at Agblangandan, Vossa, Zangnanado and Malanville (areas of low resistance) (Table 2). These knock-down values varied between 51 and 95%. At Abomey, only PermaNet 3.0 and Olyset Plus LLINs showed a knock-down effect greater than 50%.

PermaNet 3.0 was the only LLIN that showed significantly higher mortality of greater than 50% in all localities where mosquitoes carried only the *kdr* gene. The average mortality for other types of LLINs tested in these areas varied from 5 to 47% (Table 2). These mortality rates varied from 0 to 14% for Olyset, 7 to 27% for LifeNet, from 9 to 22% for Olyset Plus, from 24 to 47% for PermaNet 2.0 and from 40 to 86% for PermaNet 3.0.

### Inhibition of mortality in mono-resistant *An. gambiae* s.l. strains

The observed inhibition of mortality induced by *kdr* resistance of local mosquito strains on LLIN effectiveness was 100–86% for Olyset, 92–73% for LifeNet, 53–76% for PermaNet 2.0, 78–91% for Olyset Plus and 14–60% for PermaNet 3.0. These inhibition rates are similar to those observed with the kdr-Kisumu strain (p > 0.05).

### Knock-down (KD) and mortality induced by the LLINs on multi-resistant mosquito strains (carrying *kdr* and biochemical resistance mutations)

In areas with multi-resistance, the knock-down effects observed were also low (Table 3).

At Akron, the percentage of mosquitoes knocked down after 60 min was 31.48% [19.52–45.55] and 74.55% [60.99–85.33] for Olyset Net and Olyset Plus, respectively; 70.49% [57.43–81.84] and 81.71% [71.63–89.38] for PermaNet 2.0 and PermaNet 3.0, respectively, and 30.77% [18.71–45.1] for LifeNet. At Houéyiho, the knock-down effect was 23.08% [12.53–36.84] and 49.15% [35.89–62.5] for Olyset Net and Olyset Plus, respectively; 46.3% [32.62–60.39] and 73.5% [61.46–83.97] for PermaNet 2.0 and PermaNet 3.0, respectively, and 61.11% [46.87–74.08] for LifeNet. It was generally observed that knock-down was significantly higher with Olyset Plus than with Olyset on multi-resistant Akron and Houéyiho strains (p < 0.05). The same observation was made with PermaNet 3.0, whose knock-down was significantly higher than that observed with PermaNet 2.0.

The same observations were made at Ladji, Parakou and Tanguiéta, where the KD induced by Olyset Plus was higher than that of Olyset. Similarly, PermaNet 3.0 (98%) was more effective than PermaNet 2.0 (39%) (Table 3). However, at Tanguieta, only three LLINs were tested. The three types of mosquitoes tested showed a KD effect ≥75%. Overall, in areas where there was high activity of oxidase enzymes associated with the *kdr* gene, only three LLINs (LifeNet, Olyset Plus, and PermaNet 3.0) showed a KD effect that was generally high. However, the mortality observed in these populations was generally low (Table 3). Only the PermaNet 3.0 LLIN induced significantly higher mortality (p < 0.001) that was generally greater than 75% (Table 3).

### Inhibition of mortality in multi-resistant strains

The inhibition of the mortality induced by LLINs observed with strains carrying several resistance mechanisms (compared to the susceptible strain Kisumu) ranged from 60 to 96% for Olyset, 53 to 90.2% for LifeNet, 45 to 86% for PermaNet 2.0, 59 to 76% for Olyset Plus and 11 to 55% for Permanet 3.0. These inhibition rates are similar to those observed with the kdr-Kisumu strains (p > 0.05).

### Knock-down time of LLINs on local *An. gambiae* s.l. strains

The average time estimated for knock-down of 50% of resistant local *An. gambiae* s.l. populations was significantly shorter with PermaNet 3.0 (12 min) (p < 0.001), followed by Olyset Plus and LifeNet (33 min). However, the time required for 95% of mosquitoes to be knocked down was high for all LLINs. Generally, there was a slower effect with LLINs treated with permethrin (Table 4).

**Table 4:** Probable time for 50 and 95% knock-down of *Anopheles gambiae* s.l. per LLIN

LLINs	50% KDT (min)	95% CI	95% KDT (min)	95% CI
LifeNet	33.12	[32.5–33.91]	425.13	[385.6–468.69]
Olyset Net	98.74	[90.4–107.85]	10,257.58	[7090.39–14,839.5]
Olyset Plus	33.44	[32.56–34.34]	674.68	[595.91–763.86]
PermaNet 2.0	42.3	[41.26–43.37]	468.28	[424.57–516.49]
PermaNet3.0	12.61	[12.30–12.93]	137.99	[131.6–144.69]

*%KDT* knock down time, *IC 95%* confidence interval at 95%, *min* minutes, *CI* confidence interval

## Discussion

This study is one of the first conducted in Benin to compare the response of local malaria vectors in Benin to several LLINs recommended by the WHO. It helps to observe the variation in mortality of vectors submitted to different types of LLINs. This mortality was generally low, especially with LLINs only treated with pyrethroids. Cone tests showed that LLINs treated with piperonyl butoxide and pyrethroids (especially PermaNet 3.0) have optimum efficacy on all strains of *An. gambiae* s.l. (mono and multi-resistant).

Several studies have shown a decrease in the bio-efficacy of LLINs against local pyrethroid-resistant vectors [34, 35]. The effectiveness of LLINs treated only with deltamethrin (PermaNet 2.0 and LifeNet) was found to be significantly lower compared to that of nets treated with deltamethrin and PBO. The same observation was made with the LLINs treated with permethrin only (Olyset Net) and those treated with permethrin and PBO. However, the effectiveness of LLINs treated with permethrin was generally lower than that of LLINs treated with deltamethrin, with lower mortality and a very slow knock-down time (KDT 50 and 95%) compared to other LLINs. In a recent study conducted in Benin [36], Olyset Plus, treated with permethrin + PBO, demonstrated a higher efficacy than Olyset Net against wild multi-resistant *An. gambiae* s.l. in experimental huts, as observed in WHO cone tests used in the present study. In south-western Ethiopia [35] and in Uganda [34], a reduced efficacy of mono-treated LLINs was also observed against wild resistant *An. gambiae* s.l. in comparison with Permanet 3.0 treated with deltamethrin + PBO. The results are similar to those observed in this study. However, these studies did not include Olyset Plus, the second type of new-generation LLINs treated with permethrin + PBO.

The reduced efficacy of LLINs treated with permethrin would be related to the strong resistance of the local vectors to permethrin due to the resistance selection pressures generated by the use of the same class of insecticide for malaria vector control in public health and for pest control in agriculture [16, 17, 23, 37, 38].

The comparison of LLIN bio-efficacy performed in this study provides the necessary information for the selection of appropriate LLINs for mass distribution. The optimal and constant efficacy of PermaNet 3.0 LLINs on all vector populations shows that this combination of deltamethrin and PBO on LLINs is a most successful strategy against pyrethroid resistance in Benin. Variations in the mortality of vectors also showed that certain types of LLINs are more appropriate than others for distribution in specific regions. This is related to the fact that the effectiveness of an LLIN depends on the characteristics of the mosquito population tested and the chemical structure of the molecule (insecticide) used.

The mosquito populations assessed in the present study were characterized by a high frequency of the *kdr* gene. This high frequency was probably due to the massive use of pyrethroids in agriculture and public health. In some areas, such as Tanguieta, Parakou, Houeyiho, Akron, and Ladji, farmers and gardeners use huge amounts of insecticides to reduce pests in their crops, which explains the presence and strong expression of several resistance mechanisms in the mosquito populations [39, 40]. Over-production of resistance enzymes in these areas would be linked to pressure on mosquito larvae from insecticides used by farmers to protect vegetable crops [41–43]. This expression of the *kdr* resistance gene induced a 17–84% reduction in LLIN efficacy against laboratory strains. These frequencies are similar to those observed in natural populations of *An. gambiae* s.l. This observation shows that the *kdr* gene is the main mechanism involved in the reduction of the effectiveness of LLINs. Although detoxification enzymes contribute to resistance, their impact is successfully inhibited by the presence of PBO on new-generation LLINs and the remaining part is more likely related to the presence of *kdr* gene in the mosquito populations. This also suggests that the search for new molecules or combinations of molecules that target the *kdr* resistance mechanism should be promoted.

The WHO recommends preventive measures against vector resistance to insecticides [44]. The results of this study therefore constitute important evidence that can guide decision making in the selection and distribution of high efficacy LLINs in specific regions of Benin. The use of LLINs that showed high bio-efficacy against the local vector populations should be encouraged to contribute substantively to reducing the transmission of malaria in Benin.

This study also suggests the need to develop a routine for monitoring the bio-efficacy of LLINs against local malaria vectors for the replacement of ineffective LLINs. However, community studies would be needed to evaluate the epidemiological impact of these LLINs to confirm whether or not the low efficacy observed is followed by a loss of the epidemiological impact of these nets.

Although the important results of this study, it had certain limitations. Strong evaluation would have been possible if tunnel tests were conducted on LLINs that did not meet the criteria of 80% mortality with resistant mosquito strains. In addition, a chemical analysis of the LLINs prior to the start of the study would also have improved the quality of the results. However, all the LLINs demonstrated a good performance with susceptible laboratory stain Kisumu (mortality > 80%), as recommended by WHO [45], and the focus of this study was to demonstrate the important role of resistance mechanisms on LLINs efficacy.

## Conclusion

This study showed variable effectiveness of LLINs on *An. gambiae* s.l. populations from different localities surveyed from north to south in Benin. The new-generation LLINs with pyrethroids and PBO (PermaNet 3.0 and Olyset Plus) showed higher efficacy than conventional LLINs (PermaNet 2.0, LifeNet and Olyset net). However, the strong resistance of local vectors to permethrin suggests that the combination of deltamethrin + PBO is the most appropriate strategy against local vectors in Benin. Although the addition of PBO (targeting many biochemical mechanisms of resistance) significantly increased the mortality of mosquitoes, the significantly high role of the *kdr* resistance gene in the low efficacy of LLINs calls for LLIN technology innovation that specifically targets this mechanism.
